# Faecal proteome in clinically healthy dogs and cats: Findings in pooled faeces from 10 cats and 10 dogs

**DOI:** 10.1002/vro2.9

**Published:** 2021-04-07

**Authors:** Matteo Cerquetella, Andrea Marchegiani, Sara Mangiaterra, Giacomo Rossi, Alessandra Gavazza, Beniamino Tesei, Andrea Spaterna, Gianni Sagratini, Massimo Ricciutelli, Valeria Polzonetti, Stefania Pucciarelli, Silvia Vincenzetti

**Affiliations:** ^1^ School of Biosciences and Veterinary Medicine University of Camerino Matelica MC Italy; ^2^ School of Pharmacy University of Camerino Camerino MC Italy; ^3^ School of Biosciences and Veterinary Medicine University of Camerino Camerino MC Italy

**Keywords:** cat, dog, faecal proteomics, healthy pattern

## Abstract

**Background:**

In the scientific literature, there are only a few manuscripts available on small animal faecal proteomics.

**Methods:**

In the present pilot study, this evaluation was performed using pooled faecal samples from 10 clinically healthy dogs and, for the first time, in 10 clinically healthy cats by mean of two‐dimensional electrophoresis followed by liquid chromatography‐tandem mass spectrometry.

**Results:**

Our results showed the presence of nine (albumin, alkaline phosphatase, chymotrypsin‐C‐like, cytosol aminopeptidase, elastase‐3B/proteinase E, immunoglobulins and nuclear pore membrane glycoprotein 210) and 14 (albumin, caspase recruitment domain‐containing protein, chymotrypsin‐like, deleted in malignant brain tumours 1 protein‐like, hypothetical protein LOC107375, immunoglobulin, kallikrein‐1, superoxide dismutase, transthyretin precursor, interstitial collagenase‐like) different proteins in canine and feline faeces, respectively.

**Conclusion:**

These preliminary findings document the presence of a range of proteins in the faeces of apparently healthy dogs and cats and may serve as a basis for larger, prospective studies to establish reference proteomic data against which diseased populations can be compared.

## INTRODUCTION

Faecal proteomic analysis has been recently introduced in dogs,^1^, ^2^ with the long‐term objective of investigating new faecal markers that could be helpful in monitoring and/or diagnosing gastrointestinal (GI) diseases. Proteomics is a science aiming at analysing protein structure, function and localization in different anatomic regions.^1^, ^3^ In human medicine, the faecal proteome has been investigated in GI diseases such as colorectal cancer or other diseases like cystic fibrosis. In such cases, and in other conditions like food allergies, proteomics has been shown to be a very promising tool for finding potential markers that may be useful in diagnosing and monitoring these conditions.^3^, ^4^, ^5^ Besides, proteomics has the advantage, compared to other '‐omics' (e.g., genomics), of describing the real situation of the metabolic and functional state of a certain tissue.^3^

At the moment, only a few studies in the dog are available in the literature on faecal proteomics to draw any conclusions. In one study, the faecal proteome of healthy Boxer dogs was investigated^2^, while in another study the faecal proteome of healthy dogs was compared to that of dogs suffering from food responsive diarrhoea.^1^ In the present paper, our aim was to describe the proteins present within the faeces of a population of apparently normal dogs and cats. Besides, to the authors’ knowledge, this is the first proteomic study on feline faeces.

## MATERIALS AND METHODS

### Patients and faecal sample collection

Naturally voided faecal samples from 10 clinically healthy dogs and 10 clinically healthy cats were collected and used for the study; all owners gave informed consent. Patients were considered clinically healthy based on the absence of clinical signs of any disease at the time of sampling, absence of episodes of diarrhoea in the last 3 months, absence of diagnosis of any ongoing chronic disease (e.g., dermatopathies, endocrinopathies, cardiac disorders) according to patients’ clinical history and on the absence of any drug administered in the last 3 months (including antimicrobials, pre‐probiotics and anti‐inflammatory drugs). Additionally, all included cases were negative for intestinal parasitism based on centrifugation and flotation, Lugol staining and *Giardia* antigen testing from 3‐day pooled faecal samples.

### Sample preparation

The protein extraction was performed as described previously.^1^ Briefly, faecal samples were collected separately, immediately after production, and stored at −20°C until use. Two grams of frozen faeces from each clinically healthy animal were weighed and pooled together in order to have a total of 20.0 grams of faeces from both the healthy dogs' and cats’ groups.

The experimental design of the present work is based on the complete sample pooling strategy as previously described,^6^, ^7^ where the samples from each group of dogs and cats were pooled, and the replicates (repeated measurements of the same sample) were the technical replicates of the group.

Sixty millilitres of phosphate‐buffered saline (PBS) containing a 1:100 diluted protease inhibitor cocktail (Sigma‐Aldrich, Saint Louis, MO, USA) were added to each pooled sample. Both samples were subjected to agitation through a magnetic stirrer for 60 min on ice. Subsequently, the two mixtures were centrifuged at 10000 rpm for 20 min, at 4°C. After centrifugation, the two supernatants (from both groups) were collected, filtered three times with a paper filter, and then the resulting filtrate was subsequently filtered one more time with a 0.45 micrometres filter and one more time with a 0.22 micrometres filter (Whatman, Maidstone, UK) in order to avoid bacterial contamination from the gut microflora. To the resulting filtered samples, 90% of ammonium sulphate (Sigma‐Aldrich, Saint Louis, MO, USA) was added with slow agitation on ice, and after 30 min incubation on ice, the samples were centrifuged at 10000 rpm for 30 min at 4°C. Each resulting pellet was resuspended in 500 microliters PBS, and the total protein content was determined by the Bradford method.^8^

### Two‐dimensional electrophoresis and liquid chromatography‐tandem mass spectrometry

According to the protocol previously described by Cerquetella et al,^1^ before being loaded onto the two‐dimensional electrophoresis (2‐DE), 1 milligram of total proteins was treated with the 2‐D Clean‐Up Kit (GE‐Healthcare Life Sciences, Uppsala, Sweden). The first dimension consisted of an isoelectrofocusing in a pH range of 3–10 (Immobiline DryStrip, IPG‐strip, length 18 centimetres; IPGphor isoelectric focusing cell, GE‐Healthcare), the second dimension was a 13% SDS‐PAGE (Protean II apparatus, Bio‐Rad, Hercules, CA, USA).^1^, ^7^, ^9^ After the electrophoresis, gels were stained (0.1% Coomassie Brilliant Blue R250, 50% CH_3_OH; 10% CH_3_COOH), de‐stained (50% CH_3_OH; 10% CH_3_COOH), scanned at 600 dpi and finally subjected to image analysis using PDQuest software (Version 7.1.1; Bio‐Rad Laboratories) which allows calculation of the isoelectric point (pI), molecular mass, and normalized quantity of each spot in the gel. Among the total spots found in the 2DE maps (of both clinically healthy dogs and cats), only those showing a normalised quantity greater than 20 × 10^3^ were selected for subsequent analysis by mass spectrometry. Based on the authors’ experience, a normalised quantity value of 20 × 10^3^ is the minimum necessary to obtain a protein identification by mass spectrometry analysis. The spots obtained from both gels (clinically healthy dogs and cats) were excised, and the proteins were treated with trypsin and then extracted from the gel by following the protocol described by Shevchenko et al.^10^

After extraction, the peptides were subjected to liquid chromatography‐tandem mass spectrometry (LC‐MS/MS) performed as previously described.^1^ The resulting spectra were extracted and analysed by the MASCOT software^11^ and by the SONAR software^12^ that use mass spectrometry data for the protein identification from a peptide sequence database. For protein identification, the following search parameters were used: database, SwissProt and NCBInr; taxonomy: *Mammalia*; enzyme, trypsin; peptide tolerance, 1.2 Da; MS/MS tolerance, 0.6 Da and allowance of one missed cleavage.

## RESULTS

The mean age of dogs included was 6.5 years (minimum 2 years ‐ maximum 14 years), three were males and seven females. With regard to breeds, four were Mestizos, and one was a representative of each of the following breeds: German Shepherd, Labrador Retriever, Vizsla, Rottweiler, Weimaraner and French Bulldog. The mean age of cats was 5.5 years (minimum 2 years ‐ maximum 9 years), five were males and five females; nine were European shorthair cats, and one was a crossbreed (European shorthair cat x Persian cat).

**FIGURE 1:**
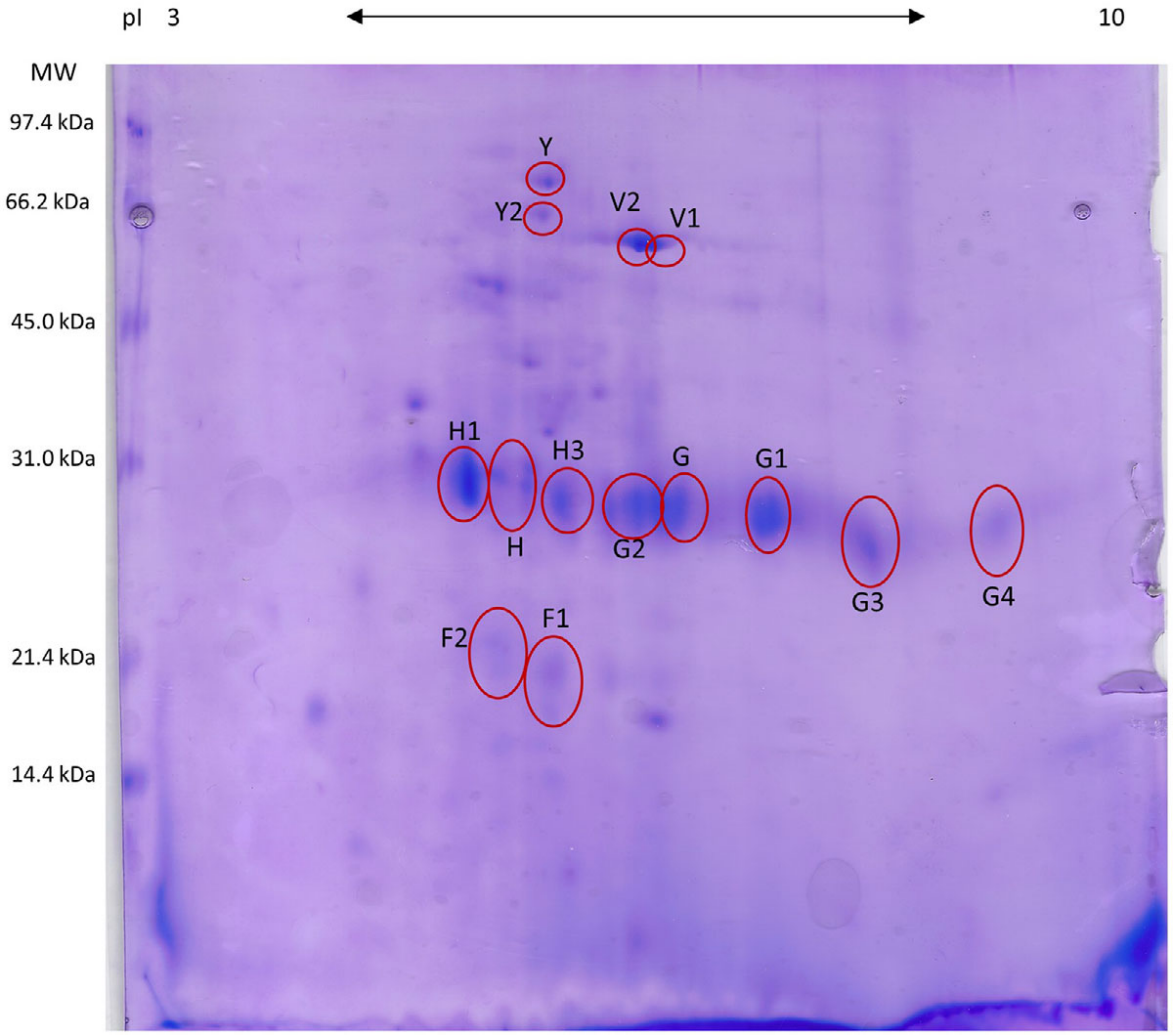
2‐DE map of the healthy dogs’ faecal proteins. The spots that were identified by the mass spectrometry are evidenced in red. The proteins were separated on an immobilized pH 3–10 linear gradient strip and subsequently subjected to a 13% SDS‐PAGE. The standards were Bio‐Rad low molecular weight (phosphorylase b, 97.4 kDa; bovine serum albumin, 66.2 kDa; ovalbumin 45.0 kDa; carbonic anhydrase, 31.0 kDa; soybean trypsin inhibitor, 21.5 kDa; lysozyme, 14.4 kDa)

**FIGURE 2:**
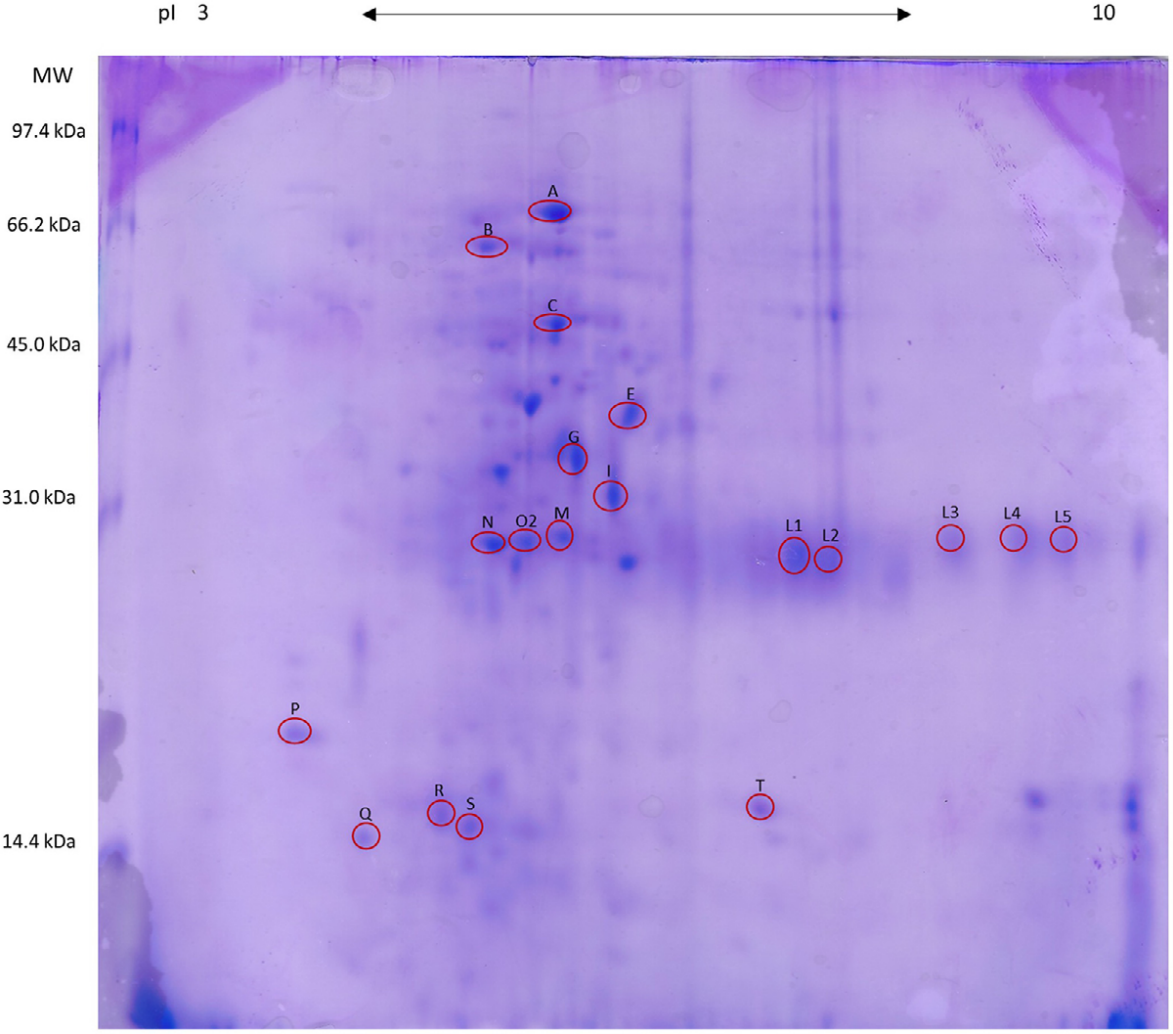
2‐DE map of the healthy cats’ faecal proteins. The spots that were identified by the mass spectrometry are evidenced in red. The proteins were separated on an immobilized pH 3–10 linear gradient strip and subsequently subjected to a 13% SDS‐PAGE. The standards were Bio‐Rad low molecular weight (phosphorylase b, 97.4 kDa; bovine serum albumin, 66.2 kDa; ovalbumin 45.0 kDa; carbonic anhydrase, 31.0 kDa; soybean trypsin inhibitor, 21.5 kDa; lysozyme, 14.4 kDa)

Results of 2‐DE evaluations for clinically healthy dogs and healthy cats are reported in Figures 1 and 2, respectively. The proteins that were identified by LC‐MS/MS followed by MASCOT and SONAR software analysis are reported in Tables 1 and 2.

**TABLE 1:** Identification of faecal proteins from healthy dogs by LC‐MS/MS followed by MASCOT^11^ and SONAR^12^ software analysis

Spot ID^a^	Protein name^b^	Score^c^	Mr (kDa)/pI^d^	Mr (kDa)/pI^e^	Sequence	Normalized quantity^e^ (x10^3^)
**Y**	Serum albumin isoform X1 (*Canis lupus familiaris*)	56	68.6/5.51	72 ± 4.6/5.8 ± 0.10	LVAAAQAALV	115 ± 47
**Y2**	Serum albumin isoform X1 (*Canis lupus familiaris*)	41	68.6/5.51	63 ± 3.0/5.8 ± 0.15	ADFAEISK	79 ± 13
**V1**	Alkaline phosphatase (*Canis lupus familiaris*)	125	68.6/6.47	59 ± 1.3/6.6 ± 0.23	ANYQTIGVSAAAR	174 ± 108
**V2**	Alkaline phosphatase (*Canis lupus familiaris*)	117	68.6/6.47	58 ± 1.6/6.6 ± 0.15	ANYQTIGVSAAAR	148 ± 51
**H**	Chymotrypsin‐C‐like (*Canis lupus dingo*)	49	29.1/5.33	29 ± 1.0/5.6 ± 0.11	LAEPVQLSDTIK	290 ± 93
**H1**	Elastase‐3B, Proteinase E (*Canis lupus familiaris*)	40	28.8/5.27	29 ± 0.8/5.2 ± 0.11	VSAFNDWIEEVMSSH	585 ± 139
**H3**	Immunoglobulin kappa light chain (*Felis catus*)	41	26.7/6.10	29 ± 1.1/6.3 ± 0.21	FSGSGSGTDFTLR	374 ± 248
**G**	Immunoglobulin λ−1 light chain (*Canis lupus familiaris*)	34	25.2/6.88	29 ± 0.9/7.1 ± 0.5	KGTHVTVLGQPK	644 ± 327
**G1**	Immunoglobulin λ−1 light chain (*Felis catus*)	39	27.8/8.17	29 ± 1.2/7.6 ± 0.6	QSNNKYAASSYL	555 ± 204
**G2**	Immunoglobulin λ‐light chain VLJ region (*Homo sapiens*)	42	29.0/8.14	29 ± 1.0/6.6 ± 0.3	EFGGGTKLTVLGQPK	642 ± 439
**G3**	Immunoglobulin λ‐light chain VLJ region (*Homo sapiens*)	30	29.0/8.14	27 ± 1.2/8.4 ± 0.5	EFGGGTKLTVLGQPK	395 ± 85
**G4**	Immunoglobulin λ‐light chain VLJ region (*Homo sapiens*)	40	29.0/8.14	27.7/8.9	QSNNKYAASSYL	285 ± 17
**F1**	Nuclear pore membrane glycoprotein 210 (*Canis lupus familiaris*)	29	192.4/6.30	19.6 ± 1.5/5.8 ± 0.14	TALLVTASISGSHAPR	227 ± 69
**F2**	Cytosol aminopeptidase (*Canis lupus familiaris*)	29	56.2/8.03	21.0 ± 1.3/5.7 ± 0.07	EILNISGPPLK	125 ± 32

**Abbreviations: Mr**, molecular mass; **pI**, isoelectric point.

**Score** number reflects the combined scores of all observed mass spectra that can be matched to amino acid sequences within that protein.

^a^ Assigned spot ID as indicated in **Figure** **1**.

^b^ MASCOT results (SwissProt and NCBInr databases).

^c^ MASCOT score reported.

^d^ From SwissProt and NCBInr databases.

^e^ Experimental values were calculated from the 2DE maps by the PDQuest software (±standard deviation).

**TABLE 2:** Identification of faecal proteins from healthy cats by LC‐MS/MS followed by MASCOT^11^ and SONAR^12^ software analysis

Spot ID^a^	Protein name^b^	Score^c^	Mr (kDa)/pI^d^	Mr (kDa)/pI^e^	Sequence	Normalized quantity^e^ (x10^3^)
**A**	Albumin (*Felis catus*)	54	68.8/5.92	68.2 ± 0.9/5.8 ± 0.15	LVNEVTEFAK	88 ± 26
**B**	Caspase recruitment domain‐containing protein 10 (*H. sapiens*)	n.d.	116/5.7	61 ± 0.5/5.3 ± 0.15	LAQLSEEK	26 ± 14
**C**	IgGFc‐binding protein (*Felis catus*)	43	318/5.28	48.4 ± 0.3/5.8 ± 0.15	LDSLVAQQLQSK	33 ± 7
**E**	Transthyretin precursor (*Felis catus*)	48	15.54/5.52	38.8 ± 0.4/6.4 ± 0.21	VLDAVQGSPAVNVAVK	52 ± 22
**G**	IgA constant region, partial (*Felis catus*)	37	42.0/5.73	34.0 ± 0.46/6.0 ± 0.15	WLQGSQELSR	16 ± 9
**I**	IgA constant region, partial (*Felis catus*)	37	42.0/5.73	31.5 ± 0.38/6.2 ± 0.15	WLQGSQELSR	47 ± 41
**L1**	Immunoglobulin kappa light chain (*Felis catus*)	48	26.7/ 6.1	28 ± 0.5/7.5 ± 0.07	GIQESTTEQNSK	n.d.
**L2**	Immunoglobulin kappa light chain (*Felis catus*)	41	26.7/ 6.1	28 ± 0.5/7.9	GIQESTTEQNSK	n.d.
**L3**	Immunoglobulin heavy chain variable region 3 (*Felis catus*)	42	21.9/4.8	27.2/8.4	DVQLVESGGDLVKPGGSLR	n.d.
**L4**	Immunoglobulin heavy chain variable region 3 (*Felis catus*)	42	21.9/4.8	28.1/8.8	DVQLVESGGDLVKPGGSLR	n.d.
**L5**	Immunoglobulin G heavy chain variable region (*Homo sapiens*)	22	13.4/6.4	n.d.	DVQLVESGGGLVQRGGSLR	n.d.
**M**	Kallikrein‐1 (*Felis catus*)	46	28.8/5.5	29.0 ± 0.35/5.9 ± 0.15	LAEPAQITDAVR	9 ± 6
**N**	Hypothetical protein LOC107375 (*Mus musculus*)	n.d.	31.7/9.7	28.0 ± 0.75/5.4 ± 0.15	YGGMLDCMASSFR	31 ± 25
**O2**	Chymotrypsin‐like elastase family member 3B (*Felis catus*)	39	28.8/5.53	28.3 ± 0.83/5.6 ± 0.15	VSAFNDWIEEV	11 ± 6
**P**	Interstitial collagenase‐like (MMP‐1) (*Felis catus*)	43	62.5/6.74	19.4 ± 1.7/4.2 ± 0.25	IENYTPDLPR	36 ± 28
**Q**	Kallikrein‐1 (*Canis familiaris*)	58	28.8/4.9	13.8 ± 1.13/4.5 ± 0.25	LAEPAQITDAVR	n.d.
**R**	Deleted in malignant brain tumours 1 protein‐like (*Felis catus*)	45	26.0/ 5.2	14.6 ± 1.9/5.1 ± 0.28	FGEGSGPIVLDDVR	9 ± 1
**S**	Deleted in malignant brain tumours 1 protein‐like (*Felis catus*)	51	26.0/ 5.2	14.6 ± 1.9/5.1 ± 0.28	FGEGSGPIVLDDVR	9 ± 1
**T**	Superoxide dismutase (Cu‐Zn) (*Felis catus*)	54	15.8/6.28	15.3 ± 1.1/7.3 ± 0.17	HVGDLGNVTAGK	55 ± 48

**Abbreviations: Mr**, molecular mass; **pI**, isoelectric point.

**Score** number reflects the combined scores of all observed mass spectra that can be matched to amino acid sequences within that protein.

^a^ Assigned spot ID as indicated in **Figure** **2**.

^b^ MASCOT results (SwissProt and NCBInr databases).

^c^ MASCOT score reported.

^d^ From SwissProt and NCBInr databases.

^e^ Experimental values were calculated from the 2DE maps by the PDQuest software (±standard deviation).

Fourteen proteins were identified for dogs (nine after excluding overlapping findings) and 19 for cats (14 after excluding overlapping findings) (Tables 1 and 2).

With regard to dogs, the spots Y and Y1 were identified as serum albumin isoform X1. Spots V1 and V2 were identified as alkaline phosphatase. Spots G and G1 were identified as immunoglobulin λ−1 light chain, and the spots G2, G3 and G4, characterised by different molecular weights and (pI), were all identified as immunoglobulin λ‐light chain, VLJ region. Other proteins identified in the dogs’ faeces are reported in Table 2.

Similarly, in cats, multiple spots on the electrophoresis gel were found to correspond to the same protein, as identified by LC‐MS/MS. They are spots G and I identified as IgA constant region, spots L1 and L2 identified as immunoglobulin kappa light chain, and spots L3 and L4 as immunoglobulin heavy chain variable region 3. Spots M and Q were recognised as kallikrein‐1, and finally, spots R and S identified as deleted in malignant brain tumours 1 protein‐like (DMBT‐1). Other proteins identified in the faeces of cats are shown in Table 2.

It can be noted (Tables 1 and 2) that in some cases the identified proteins have been attributed to a different animal species. In fact, in dogs, eight proteins were ascribed to *Canis lupus familiaris*, while two to *Felis catus*, three to *Homo sapiens*, and one to *Canis lupus dingo*, whereas in cats 15 spots were ascribed to *Felis catus*, while two to *Homo sapiens*, one to *Canis lupus familiaris*, and one to *Mus musculus*. Since a specific taxonomy *Canis lupus familiaris* is not available in the database, in this analysis the taxonomy *Mammalia* has been used; therefore it could be possible that a protein is assigned to a different species. Furthermore, it should be noticed that the more peptides MASCOT identifies from a protein, the higher is the MASCOT score for that protein; in this study, proteins were identified with high MASCOT scores. In addition, if a sequence alignment of the identified proteins from different species is carried out, their high sequence homology with those from *Canis lupus familiaris* were observed (e.g., immunoglobulin kappa light chain from *Canis lupus familiaris* and *Felis catus* has 82.7% sequence homology), thus suggesting the reliability of the protein identification.

## DISCUSSION

2‐DE is a powerful tool that allows the separation, identification and quantification of proteins in complex mixtures when coupled with mass spectrometric analysis. This technique finds a particular application in the clinical field as it may help to provide pathophysiological insight, to identify proteins associated with certain pathologies and to aid in the identification of new drug targets and diagnostic and prognostic disease biomarkers.^13^ In particular, the aim of clinical proteomics is the detection of new drug targets and diagnostic and prognostic disease biomarkers.^14^ Proteomics also finds applications in the field of veterinary medicine, with particular emphasis on infectious diseases, animal diseases pathogenesis, veterinary diagnostics, as demonstrated by the numerous works that can be found in the literature.^15^, ^16^ The aim of the present work was the proteomic analysis of faeces from a population of apparently healthy dogs and cats. These data may serve as a basis for larger, prospective studies to establish a reference faecal proteome from normal animals. With the present study, we found nine different proteins in dog faeces and 14 in feline faeces. Only three proteins (or similar proteins) were identified in both dogs and cats: albumin (spot A) in cats and serum albumin isoform X1 (spot Y) in dogs, chymotrypsin‐like elastase family member 3B (spot O2) in cats and chymotrypsin‐C‐like (spot H) in dogs and, finally, immunoglobulin kappa light chain in both cats (spots L1 and L2) and dogs (spot H3). When looking for proteins (SwissProt & NCBInr databases, plus the CanisOme database^17^) that had been previously found in canine faeces we only found one, the immunoglobulin λ−1 light chain that partially overlaps with a protein found in a study of healthy Boxer dogs, although this previous study referred to the protein as immunoglobulin lambda‐1 light chain isoform X36.^2^ The fact that this is only a single partially similar result may be due to the use of different criteria for spots selection and that the present study did not include Boxers. This difference suggests that other factors, such as breed, may impact on the faecal proteome and more studies on a larger number of animals, divided by breed, are therefore required to investigate this further.

Other proteins that were found in this study included albumin (spots Y and Y2) and enzymes (spots V1, V2, H, H1 and F2). Reasons for these findings could include physiological enterocyte turnover (and associated cell death) and/or physiological enzymatic secretion associated with digestive processes, as both can lead to the release of intracellular content into the intestinal lumen. Examples of such an occurrence include when the protease elastase‐3B (spot H1) is found in the human small intestine,^18^ and when alkaline phosphatase (spots V1 and V2) has been found in faeces of healthy dogs.^19^ It is also easy to explain the presence of immunoglobulins (spots H3, G, G1, G2, G3 and G4) as the result of a physiological activation of the immune system. The presence of multiple forms of the immunoglobulin λ−1 light chain, VLJ region (spots G2, G3, G4), may be a consequence of the rearrangements that the variable region of the immunoglobulin heavy and light chains undergoes in order to generate antibody diversity in response to and antigen stimulus. On the other hand, the importance of identification of the nuclear pore membrane glycoprotein 210, (spot F1), a transmembrane protein component of metazoans nuclear pores with unknown function, is challenging to ascertain.^20^

No previous studies on the feline proteome have been performed for comparison. However, if we also look for the causes of protein presence in this group, the retrieval of albumin (spot A), enzymes (spots B, M, O2, P, Q and T) and immunoglobulins (spots C, G, I, L1, L2, L3, L4 and L5) can be explained as previously reported in the dog. The presence of superoxide dismutase [Cu‐Zn] (spot T), an enzymatic antioxidant expressed by almost all mammalian cells and found in cytosol, mitochondria and extracellular surfaces can be explained as a result of physiological mucosal turnover.^21^ With regard to transthyretin precursor (spot E), it is a protein secreted from the liver and choroid plexus into the blood and the cerebrospinal fluid, respectively. It is a transporter for thyroid hormones and a retinol binding protein with very modest proteolytic activity towards very few substrates.^22^ In human medicine, a reduction in plasma transthyretin could be related to severe acute phase response to thyroid diseases, but in veterinary medicine its role is not completely elucidated.^23^ The few studies performed in animals have shown a lower concentration of transthyretin in 1 month diarrheic calves compared with healthy controls and reduced plasma concentrations in *Mycobacterium avium paratuberculosis* seropositive cows compared to healthy cattle.^23^ Further studies are needed to understand the possible reason(s) behind the presence of transthyretin precursor in the faeces of clinically healthy cats. Conversely, DMBT1 protein‐like (spots R and S) is a member of the superfamily cysteine‐rich scavenger receptor. This protein is involved in epithelial differentiation, innate immunity and inflammation, and it is produced also by mucosal cells of the GI tract, where it exerts protective effects on the epithelium; therefore its presence in faeces can be justified.^24^, ^25^ Lastly, the presence of spot N (hypothetical protein LOC107375) is impossible to interpret, due to the lack of a precise correspondence.

Limitations of the present pilot study include the enrolment of a small number dogs and cats, the absence in inclusion criteria of complete clinicopathological testing or diagnostic imaging to exclude any possible underlying disease and the absence of diet standardisation. Unfortunately, considering the almost total absence of similar studies in the scientific literature, it is impossible to state if, and in case to what extent, individual variations, breed, diet, age, etc. could influence the faecal proteome. Although a sample pooling strategy was used to minimise biological variation, unfortunately this resulted in an inability to evaluate the effect of individual variation, or differences associated with age, breed or diet. Consequently, in the absence of studies to investigate the impact of these factors, it is possible that individual proteome would have differed considerably between subjects. Furthermore, given the small sample size, the list of identified proteins may not be exhaustive. Even though such aspects need to be further addressed in future studies, we believe that the present data represent a first step towards the identification of the faecal proteome in clinically healthy dogs and, for the first time, cats.

## CONCLUSION

The present pilot study demonstrates the presence of a range of proteins in the faeces of apparently healthy dogs and cats, as determined by 2‐DE and LC‐MS/MS. These findings may serve as a basis for larger, prospective studies to clarify the degree of normal individual variation, the effects of diet and other confounding factors and potentially to establish reference proteomic data against which diseased populations can be compared.

## AUTHOR CONTRIBUTIONS

Cerquetella Matteo and Vincenzetti Silvia conceived and designed the study. Cerquetella Matteo, Mangiaterra Sara, Ricciutelli Massimo, Pucciarelli Stefania and Vincenzetti Silvia performed the fieldwork, laboratory and statistical analysis. Cerquetella Matteo, Marchegiani Andrea, Ricciutelli Massimo and Vincenzetti Silvia wrote the paper. Rossi Giacomo, Gavazza Alessandra, Tesei Beniamino, Spaterna Andrea, Sagratini Gianni and Vincenzetti Silvia advised on study design and help in results interpretation. All authors contributed to the revision of the article and to the writing in its current form and approved the final version.
